# Turbocharging protein binding site prediction with geometric attention, inter-resolution transfer learning, and homology-based augmentation

**DOI:** 10.1186/s12859-024-05923-2

**Published:** 2024-09-20

**Authors:** Daeseok Lee, Wonjun Hwang, Jeunghyun Byun, Bonggun Shin

**Affiliations:** 1 Deargen, Seoul, Republic of Korea; 2 SK Life Science, Inc., Paramus, NJ USA

**Keywords:** Protein binding site prediction, Ligand agnostic binding site prediction, SE(3) invariant and equivariant deep learning, 3D convolutional neural networks, Transfer learning, Protein homology

## Abstract

**Background:**

Locating small molecule binding sites in target proteins, in the resolution of either pocket or residue, is critical in many drug-discovery scenarios. Since it is not always easy to find such binding sites using conventional methods, different deep learning methods to predict binding sites out of protein structures have been developed in recent years. The existing deep learning based methods have several limitations, including (1) the inefficiency of the CNN-only architecture, (2) loss of information due to excessive post-processing, and (3) the under-utilization of available data sources.

**Methods:**

We present a new model architecture and training method that resolves the aforementioned problems. First, by layering geometric self-attention units on top of residue-level 3D CNN outputs, our model overcomes the problems of CNN-only architectures. Second, by configuring the fundamental units of computation as residues and pockets instead of voxels, our method reduced the information loss from post-processing. Lastly, by employing inter-resolution transfer learning and homology-based augmentation, our method maximizes the utilization of available data sources to a significant extent.

**Results:**

The proposed method significantly outperformed all state-of-the-art baselines regarding both resolutions—pocket and residue. An ablation study demonstrated the indispensability of our proposed architecture, as well as transfer learning and homology-based augmentation, for achieving optimal performance. We further scrutinized our model’s performance through a case study involving human serum albumin, which demonstrated our model’s superior capability in identifying multiple binding sites of the protein, outperforming the existing methods.

**Conclusions:**

We believe that our contribution to the literature is twofold. Firstly, we introduce a novel computational method for binding site prediction with practical applications, substantiated by its strong performance across diverse benchmarks and case studies. Secondly, the innovative aspects in our method— specifically, the design of the model architecture, inter-resolution transfer learning, and homology-based augmentation—would serve as useful components for future work.

## Background

In structure-based drug discovery, the knowledge of ligand binding sites (hereafter binding sites) on target proteins is crucial. It aids *rational drug design* [12, 27, 36] and is required for *in-silico* methods such as docking [3, 27]. Such knowledge of binding sites can be attained if the crystal structure of the ligand-bound target protein is available. However, if no such structure is available, one may rely on computational means to identify the binding sites.

### Sub-tasks

In general, this computational task of *binding site prediction (BSP)* can be regarded as a composition of two sub-tasks: (1) *binding site detection (BSD)* and (2) *binding residue identification (BRI)*.

Firstly, BSD aims to identify the binding sites in a coarse-grained manner and score their druggability. Successful detection of highly druggable binding sites can aid medicinal chemists in many ways when designing better drug compounds. For example, medicinal chemists can draw valuable insights into improving drug compounds’ binding affinity or selectivity by examining the receptor structure at the potential binding site [12]. Also, preparing a suitable binding site constitutes the first step in any in-silico structure-based drug discovery pipeline [3].

Secondly, BRI aims to identify residues in a given binding site that play key roles in interactions with ligands. Identification of such critical residues has been pursued in many previous research papers due to its importance in rational drug design [5, 27, 36]. In particular, it has several applications in in-silico structure-based drug discovery. For example, *structural pharmacophore* features can be selected based on the identified key residues [2, 27], and docking results can be prioritized according to whether the docked molecule has favored interactions with the key residues [3, 27].

### Existing methods

In the following, we discuss (1) traditional methods for BSP, (2) structure-based deep learning methods for BSP, and (3) methods tackling a similar problem of predicting ligand-specific binding sites.

#### Probe-based methods

These methods use a fixed set of small molecules called “probes” to determine the binding sites in a query protein [14, 20, 26]. Specifically, they place the probes at different positions on the protein’s surface and calculate the physical energy at the positions. The low-energy positions are predicted to be potential binding sites.

#### Geometry-based methods

These methods rely on 3D geometric characterization of binding sites to detect them. [4, 21, 46]

One example is Fpocket [21], which tries to find concave regions of appropriate sizes on protein surfaces. It does so by approximating the local curvatures by radii of alpha spheres, which are spheres with four heavy atoms on them but no heavy atom inside. More specifically, it finds all alpha spheres within a radius range, clusters them, and filters them according to the number of constituent alpha spheres to produce a binding pocket.

Although Fpocket typically produces an excessive number of binding pockets, it has a relatively good recall (96.4% on scPDB v.2017, according to [11]. Therefore, there are ML-based algorithms [1, 19] that utilize Fpocket as a means to generate initial candidate binding sites.

#### Template-based methods

These methods predict the binding sites of a given protein by leveraging templates, which are proteins with known binding sites that share similarities with the query protein [32, 33, 41, 42]. A portion of the query protein is regarded as a binding site if it resembles binding sites of templates either sequentially or structurally.

For example, the authors of [42] suggested combining two template-based approaches, one based on substructure comparison (TM-SITE) and the other on sequence profile alignment (S-SITE). TM-SITE works as follows:

1. Putative binding pockets are identified in the query protein by relying on external software [7].
2. For each putative binding pocket, similar template binding sites are collected as putative templates. The similarity measure is based on both structural and sequential comparisons.
3. The ligands in the putative templates are projected to the binding pocket.
4. The residues in the binding pocket are determined to be in the binding site if they are close enough to the majority of the projected ligands.

On the other hand, S-SITE works as follows:

1. The query protein sequence is aligned with the template sequences based on their position-specific scoring matrices (PSSM) profiles and secondary structure information.
2. Templates with the highest alignment *quality scores* are chosen as the putative templates.
3. The residues in the query sequence are determined to be in the binding site if they are aligned with the majority of the putative templates.

#### Structure-based deep learning methods

*DeepSite* DeepSite[16] predicts the binding sites by using a 3D CNN model and a clustering algorithm. The inference steps of DeepSite are as follows: it (1) generates points spanning the entire protein-occupied 3D space, (2) predicts the *ligandability* of the points using the CNN model computed on a 3D grid centered at the points, and (3) clusters the ligandable points to produce binding sites.

*DeepSurf* DeepSurf[25] has an overall procedure similar to DeepSite[25] but uses more sophisticated approaches in several aspects. More specifically, it tries to improve (1) the generation of initial points, (2) the formation of input grids, and (3) the architecture of the 3D CNN model. The initial points are sampled on the solvent accessible surface (SAS) of the protein rather than the entire span of the protein. Then, the axes of the grids formed at those points are not arbitrarily oriented, but one axis is set to be the normal vector of the SAS. Finally, rather than using a plain CNN model, they used 3D equivalents of ResNet and bottleneck ResNet [13].

*Kalasanty* Kalasanty [35] approaches binding site prediction (BSP) by framing it as a 3D *image segmentation* problem. Therefore, it uses a 3D equivalent of the U-net model [31], originally developed for 2D images. It applies the U-net model to large grids covering most of the query proteins. Then, it outputs the connected components consisting of positively predicted voxels as binding sites.

*Deeppocket* Deeppocket [1] has separate detection and segmentation models, both of which rely on the binding site candidates generated by Fpocket. The detection model is a plain 3D CNN model, and the segmentation model is a U-net model similar to the one used in Kalasanty. The former is used to rank the binding site candidates generated by Fpocket, and the latter is used to segment the 3D voxels centered at the top-ranked sites.

#### Predicting ligand-specific binding sites

Recently, deep learning models that predict protein-ligand complex structures, given a protein-ligand pair, have been developed [22, 24, 34]. In principle, these models can be used to find ligand-specific binding sites. Therefore, one may argue that the BSP models are strictly less useful than these models, since their predictions on binding sites do not take into account the partner ligands. However, we argue that they are still useful in their own right. Firstly, for many applications, the prediction of ligand-agnostic binding sites is not only sufficient but also desirable. For example, a typical docking experiment requires a binding pocket location as a prerequisite and docks all molecules in a virtual library to the pocket. To predict binding pockets using the models that do consider the partner ligands, preparing appropriate ligands may add additional complexity to the problem. This is similar to the problem of preparing appropriate *probe* molecules in the previously mentioned *probe-based methods*. Secondly, the performances of the methods that predict protein-ligand complex structures are not satisfactory at this point. For example, [6] demonstrated that recent deep learning methods [10, 22, 23, 34, 45] exhibited inferior performance compared to Vina [37], especially when evaluated on proteins whose sequence identity to the training set is low. Moreover, most of the poses that were predicted by these methods were chemically or geometrically invalid. Hence, it might be prudent to concentrate on the more manageable and well-studied BSP problem.

### Focus of the study

In this paper, we will focus in particular on the structure-based deep learning methods to tackle the BSP problem. This choice reflects two recent trends. Firstly, deep learning has been widely adopted for BSP [44], and has shown notable performance. Secondly, it has become easier to identify protein structures as a result of (1) the rapidly accumulating experimental data in databases such as PDB, and (2) advancements in deep learning methods, exemplified by Alphafold [18, 38].

### Limitations of existing methods

The recent structure-based deep learning methods for BSP have several problems that limit the performance in BSD and BRI.

Firstly, the CNN-only architecture may be inefficient in encoding long-range patterns. Since a convolution layer’s operation is only local, a deep hierarchy of convolutions must be applied to allow a neuron to have a receptive field large enough to cover the global patterns. This long-term dependency is known to impede training [15]. The large CNN models used in Kalasanty and the segmentation model of Deeppocket may suffer from this problem, while small CNN models used in other models would require additional sub-optimal mechanisms such as clustering, as in DeepSite and DeepSurf.

Secondly, the grid-based models require excessive post-processing to interpret the outputs to the desired level (either pocket or residue). For the prediction of binding pockets, they (excluding Deeppocket) employ clustering on high-probability points, either voxels or grid centers, to delineate pockets. For the prediction of binding residues, they posit that an atom close to a high-probability point is part of a binding residue. This may lead to sub-optimal BSD and BRI performances, since the loss function used to train the model does not compare the ground truth with the final output but with the intermediate output before post-processing. To achieve better performance, it would be preferable to have neural networks that directly generate outputs at the desired levels.

Lastly, the existing methods do not fully utilize existing data sources. These data sources include (1) the information at the finer resolution and (2) the large unlabelled database of protein structures, which recently became available due to [38]. An example of a prominent method lacking the former data source is the detection model of Deeppocket, which still recorded high performance compared to other state-of-the-art methods. It essentially ignored the shape of the binding site and only retained the pocket-level label during training. Next, the latter data source has not been utilized in BSP methods, to the best of our knowledge. The extensive unlabeled database holds immense potential to advance state-of-the-art performance, as evidenced by progress in other domains of deep learning [39, 43].

### Aim of the study

In this study, our objective is to formulate a new model architecture along with its training method to address all the aforementioned challenges.

## Problem definition

Thus far, the BSP task has not been addressed explicitly under a common definition across different literatures, even though they used different model compositions. For example, while Deeppocket is comprised of separate “detection” and “segmentation” models, Kalasanty only uses a single segmentation model, whose output is post-processed by a clustering algorithm.

In order to fairly assess different BSP models, it is necessary to envisage a unified definition of the BSP task. To be more specific, we will formally establish standards for the input and output. All baseline models can be regarded as following the standard, which will be explained in the experiment section.

Moreover, we will also explain the decomposition of the task into sub-tasks (including BSD and BRI), which is employed in our method and Deeppocket.

### The BSP task

BSP is the task of identifying the ligand binding sites in a given protein. In the task, we are given a protein structure *P* and the number of binding sites *n* as input. We assume that there are known structures of ligands $l_i$ ($i=1,\cdots ,n$) that correspond to the binding sites.

The goal of the task is to predict an unordered set of *n* binding sites of *P* where the ligands $l_1,\cdots ,l_n$ bind.

A *predicted binding site* is of the form $({\hat{c}}_i, {\hat{R}}_i)$, where ${\hat{c}}_i\in \mathbb {R}^3$ is the *binding site center* and ${\hat{R}}_i\subset \left\{ 1,\cdots ,size(P)\right\}$ is the set of indices of *binding residues*. For example, an ideal prediction $\left\{ ({\hat{c}}_1,{\hat{R}}_1),\cdots ,({\hat{c}}_n,{\hat{R}}_n)\right\}$ is such that

- ${\hat{c}}_i$ is close (e.g. within the radius threshold $4\mathop {\textrm{A}}\limits ^{\circ }$) to $l_i$
- ${\hat{R}}_i$ is the set of indices of residues close (e.g. within the radius threshold $4\mathop {\textrm{A}}\limits ^{\circ }$) to $l_i$

The methods we used to evaluate such predictions will be explained in "Evaluation methods" section.

Note that the use of binding residues as a representation of the shape of the binding sites is neither definitive nor universal, but it is our choice because we see many applications of this approach as explained in 1.1.

### Decomposition into sub-tasks

Our method divides the BSP task into sub-tasks (1) candidate generation, (2) Binding Site Detection (BSD) and (3) Binding Residue Identification (BRI), each corresponding to a dedicated module (this is similar to TM-SITE [42] and Deeppocket [1]). To be more specific, let (*P*, *n*) be an input to perform BSP on. First, the *candidate generation module* takes the protein structure *P* as an input, and then generates the candidate binding site centers ${\hat{c}}'_1, {\hat{c}}'_2,\ldots , {\hat{c}}'_m\in \mathbb {R}^3$, where typically $m \gg n$. Next, the *BSD module* takes $(P, {\hat{c}}_i)$ ($1\le i\le m$) as the inputs, and then outputs the predicted *druggability* of ${\hat{c}}'_i$ in *P*. The druggability scores are then used to rank the candidate centers, the top *n* of which form a filtered list ${\hat{c}}_1,\cdots ,{\hat{c}}_n$ of candidate centers. Lastly, for each $1\le i\le n$, the *BRI module* takes as input $(P, {\hat{c}}_i)$, and outputs ${\hat{R}}_i$, that is the set of binding residues within the binding site. The resulting set $\left\{ ({\hat{c}}_1, {\hat{R}}_1),\cdots ,({\hat{c}}_n,{\hat{R}}_n)\right\}$ becomes the final output of the model.

**Fig. 1:**
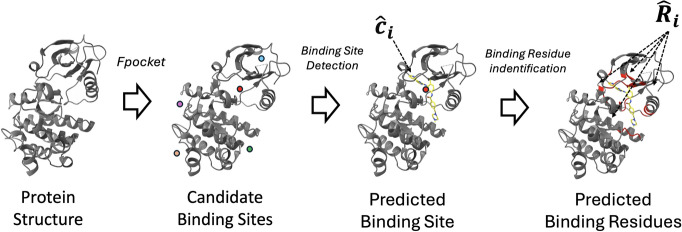
This figure illustrates the overall inference procedure of our method. This reflects the decomposition of BSP into sub-tasks described in "Decomposition into sub-tasks" section

## Methods

**Fig. 2:**
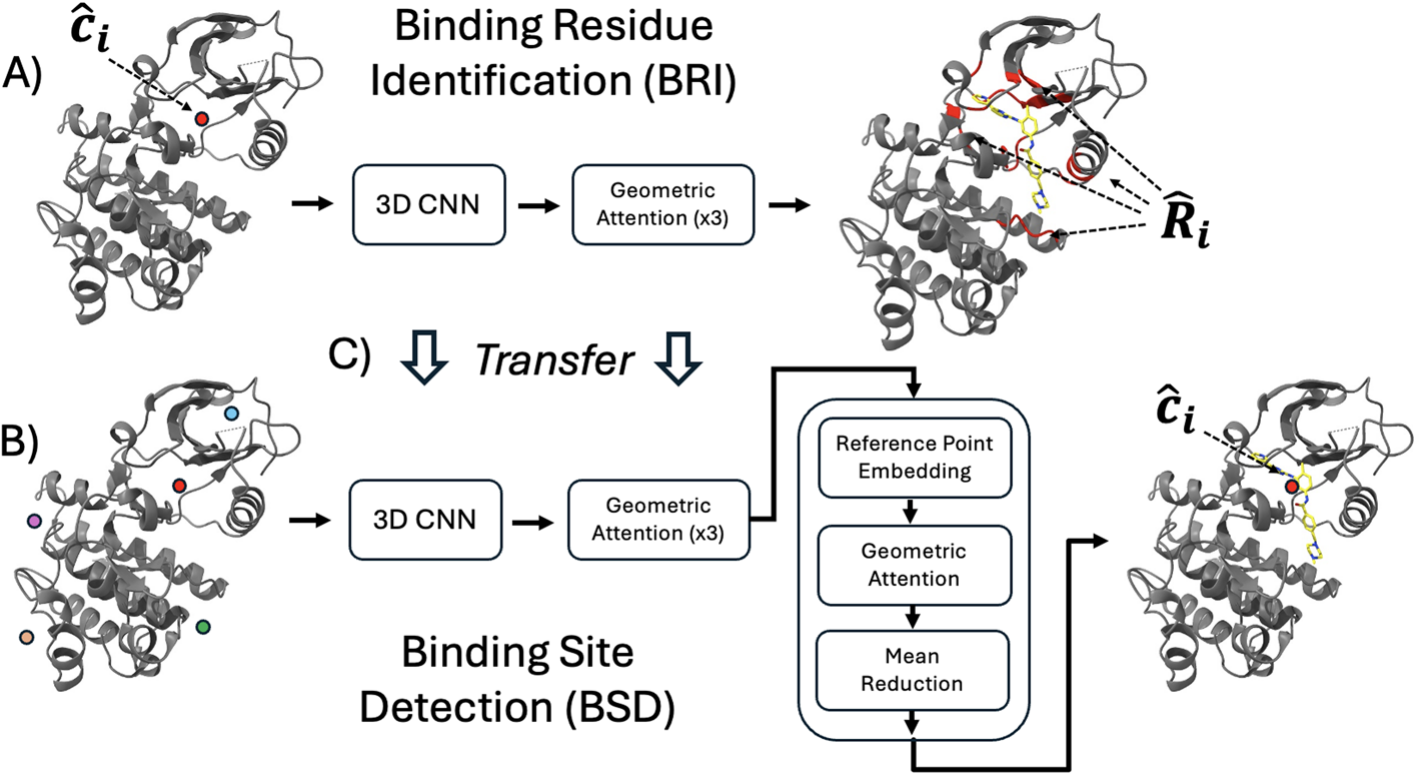
This figure illustrates the architectures of our BRI and BSD modules and the training procedure. The roles of these modules within the prediction procedure are explained in "Decomposition into sub-tasks" section and Fig. 1. (**A**) Our BRI module utilizes a 3D CNN and geometric attention layers to score each candidate residue within radius $17\mathop {\textrm{A}}\limits ^{\circ }$. A more detailed illustration of the architecture is provided in "The BRI module" section. For inference, residues with scores higher than 0 (sigmoid probability 0.5) constitute the predicted binding residues $\hat{R_i}$. For training, the scores are compared with the true binding residues determined by the condition that any non-hydrogen atom of it is within $4\mathop {\textrm{A}}\limits ^{\circ }$ from any ligand non-hydrogen atom. Note that this $4\mathop {\textrm{A}}\limits ^{\circ }$ condition was used in [1] and [25] to find binding site residues and atoms respectively. More technical details of the training are provided in "Training the BSD module" section. (**B**) Our BSD module utilizes the same backbone architecture as the BRI module but is followed by additional layers to produce outputs on the level of candidate binding sites. A more detailed illustration of the architecture is provided in "The BSD module" section. For inference, *n* top-scored candidate binding sites constitute the predicted binding sites $\left\{ \hat{c_i}\right\} _{i=1}^{n}$. For training, the scores are compared with the true binding sites determined by the condition that they are within $4\mathop {\textrm{A}}\limits ^{\circ }$ from any ligand non-hydrogen atom (i.e. Distance center to atom (*DCA*) is smaller than $4\mathop {\textrm{A}}\limits ^{\circ }$) following [1] and [25]. More technical details of the training are provided in "Training the BRI module". (**C**) Our BSD module is trained in two stages, where in the second stage, the parameters of the parts shared with the BRI module are initialized from the result of training a BRI module in the first stage

### The model architecture

#### The candidate generation module

To generate the binding site candidate centers, we use an external software Fpocket [21]. Given a protein structure, Fpocket finds sets of heavy atoms ${\hat{S}}_1,\ldots , {\hat{S}}_m$, each corresponding to a region geometrically likely to be a binding pocket. Then, we find the candidate centers ${\hat{c}}'_i$ ($i=1,\ldots ,m$) by taking the center of the mass of the atoms in ${\hat{S}}_i$.

We chose Fpocket as the candidate generation method because it achieves a sufficiently high recall rate (96.4% on scPDB v.2017, according to [11]. This means that, for a given protein and its binding site, it is likely that at least one of the generated candidates corresponds to the binding site. Then, provided that the BSD module ranks the candidates properly, the top-*n* candidates may approximate the true binding site centers with a high accuracy.

#### The BSD module

The BSD module takes as input the protein structure and a candidate binding site center ${\hat{c}}'$ and outputs the predicted druggability at ${\hat{c}}'$.

In this process, it featurizes the surroundings of ${\hat{c}}'$ into a set of per-residue 3D grids, and processes the grids through a neural network to produce the output feature. Here, each grid in the set corresponds to a residue close enough to ${\hat{c}}'$ (distance threshold $17\mathop {\textrm{A}}\limits ^{\circ }$), and encodes the local environment of the residue. Note that the distance threshold $17\mathop {\textrm{A}}\limits ^{\circ }$ is justified in Section 5 of Supplementary Information. In short, (1) it is at about the same level as the input size of Deeppocket’s segmentation model in a sense and (2) there was a consideration of computational resources.

The neural network of the BSD module is composed of (1) a residue-local feature extraction unit that runs in parallel for each grid, (2) an aggregation unit that globally aggregates the local features, and (3) a reduction unit that maps the aggregated feature to a single scalar quantity. The feature extraction unit is a 3D CNN model, and the aggregation unit is composed of several geometric self-attention layers. The reduction unit is composed of a point-wise feed-forward layer and a mean-reduction operation.

#### The BRI module

The BRI module takes in the protein structure and a putative binding site ${\hat{c}}$ as inputs and outputs the set of predicted binding residue indices.

The BRI module shares the residue-local feature extraction and global aggregation units with the BSD module. To be more specific, the BRI module shares the following units with the BSD module: (1) the CNN feature extractor and (2) the stack of geometric attention layers up to the penultimate one in the BSD module. However, the remaining part of the BRI module is only comprised of a point-wise feed-forward layer without a mean-reduction operation. Consequently, the outputs of the last layer are used to determine (with the threshold 0) whether the corresponding residues are binding site residues or not.

#### The CNN model

For the CNN architecture, our BSD and BRI modules use a 3D bottleneck ResNet model adopted in [25]. The model is adapted from the bottleneck ResNet model introduced in [13] for image classification. The bottleneck architecture reduces the number of parameters, thereby facilitating the employment of a deeper network. [25] demonstrated that the 3D bottleneck ResNet model, despite its lightweight design, achieved competitive performance in comparison to its non-bottleneck counterpart.

#### The geometric attention layers

For the geometric attention layers, our BSD and BRI modules use an attention mechanism introduced in [18], with a slight adjustment necessary to accommodate the forms of inputs.

The inputs of the attention layers are composed of the following:

- $x_i\in \mathbb {R}^{d_{hidden}}$ ($i=1,\cdots ,n$), hidden vectors associated with the residues.
- $T_i=(R_i, t_i)\in SO(3)\times \mathbb {R}^3$ ($i=1,\cdots ,n$), the *local frames* associated to the residues, where $t_i$ is the position of the alpha carbon and $R_i$ is the rotation matrix that represents the residue orientation (See 1.3 in Supplementary Information). Note that the operation $v\mapsto T_i v$ maps the local coordinates (concerning the local frame) to the corresponding global coordinates, and the operation $u\mapsto T_i^{-1} u$ does the reverse.

Then, the computation is carried out in the following steps:

1. The standard query and key vectors $q_i^h$ and $k_i^h$ are computed by the linear mappings from $x_i$. Here, *h* denotes a “head”.
2. The geometric query and key vectors ${\textbf {q}}_i^{hp}$ and ${\textbf {k}}_i^{hp}$ in $\mathbb {R}^3$ are computed by the linear mappings from $x_i$. Here, *h* denotes a “head” and *p* denotes a “point” of attention.
3. The *attention weight* from the *i*-th token to the *j*-th token is computed from a linear combination of the standard attention weight
  3.1 $$\begin{aligned} w_{ij}^{h,standard }=\frac{1}{\sqrt{d_{hidden}}}q_i^h\cdot k_j^h \end{aligned}$$
  and the geometric attention weight
  3.2 $$\begin{aligned} w_{ij}^{h,geometric}=\frac{1}{\sqrt{N_{points}}}\sum _{p}\left\| T_i{\textbf {q}}_i^{hp} -T_j{\textbf {k}}_j^{hp}\right\| \end{aligned}$$
  by applying a softmax operation. More precisely, the attention weight becomes
  3.3 $$\begin{aligned} w^h_{ij} = softmax_{j} \left(\frac{1}{\sqrt{2}}\left(w_{ij}^{h,standard}-\log \left(1+\gamma ^h\right)w_{ij}^{h,geometric}\right)\right) \end{aligned}$$
  where $\gamma ^h$ is a learnable parameter.
4. The standard value vectors $v_j^h$ are computed by a linear map from $x_j$, and aggregated as
  3.4 $$\begin{aligned} o_i^h=\sum _{j}w_{ij}^hv_j^h \end{aligned}$$
5. The geometric value vectors ${\textbf {v}}_j^h$ are computed by a linear map from $x_j$, and aggregated as
  3.5 $$\begin{aligned} {\textbf {o}}_i^{hp} = T_i^{-1} \left( \sum _{j}w_{ij}^hT_j{\textbf {v}}_j^{hp}\right) \end{aligned}$$
6. The aggregated vectors as well as their sizes are concatenated and linearly mapped via $f_{final}$ to produce the output of the attention layer
  3.6 $$\begin{aligned} x_i'= f_{final}\left(concat_{h,p}\left(o_i^h,{\textbf {o}}_i^{hp},\left\| {\textbf {o}}_i^{hp}\right\| \right)\right) \end{aligned}$$

Note that the adjustment made to the original attention mechanism is the omission of the “attention bias” term.

### The inter-resolution transfer learning

As illustrated in Fig. 2, transfer learning can be applied between the BSD and BRI sub-tasks, taking advantage of the shared architectures between the BSD and BRI modules. More specifically, we initialize the weights of the BSD module’s shared parameters with the weights obtained from the training of the BRI module. The rationale behind this procedure is the following intuition: the protein’s binding site can be determined based on the patterns of the binding residues.

### The homology-based augmentation

**Fig. 3:**
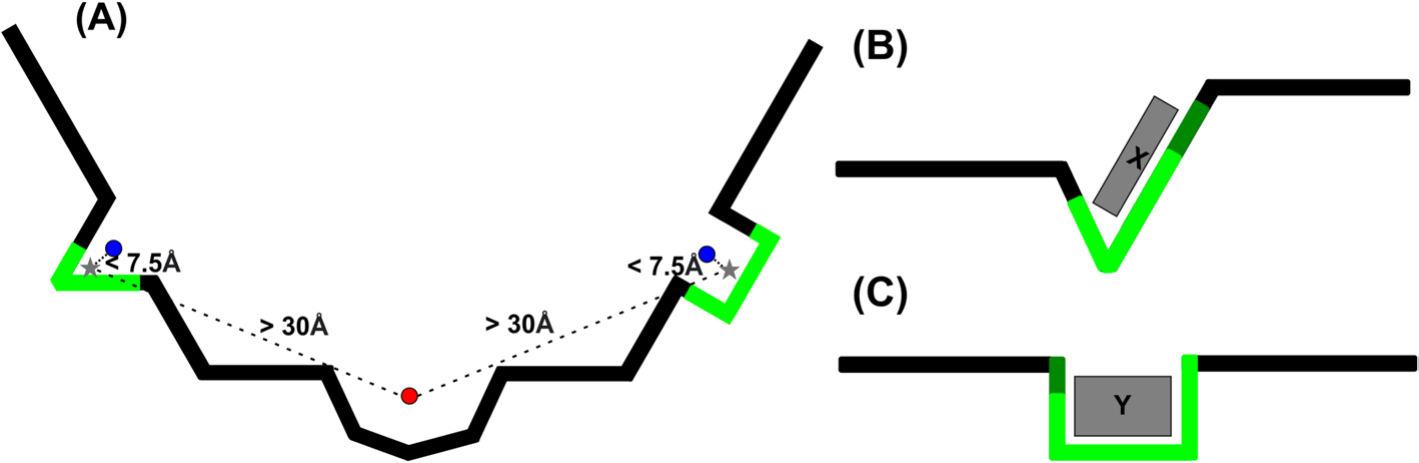
These figures illustrate how our homology-based augmentation determines the positive and negative binding site candidates in augmented proteins. **A** Depicts an augmented protein with two positive (the blue points) and one negative (the red point) binding site candidate centers. Among the binding site candidates proposed by Fpocket, they are labeled based on the distances to the *proxy centers* (the star-shaped points) of binding sites inferred from homology relations. **B** and **C** depict the homologous proteins in the original database that contributed to the inferred binding sites in the augmented protein. *X* and *Y* are their ligands. The bright and dark green regions of the chains indicate the residues in close proximity to the ligands, while only the bright green region exhibits evolutionary correspondence to residues in the augmented protein. The bright green region must comprise at least 50% of the entire green region for the binding site to count

**Fig. 4:**
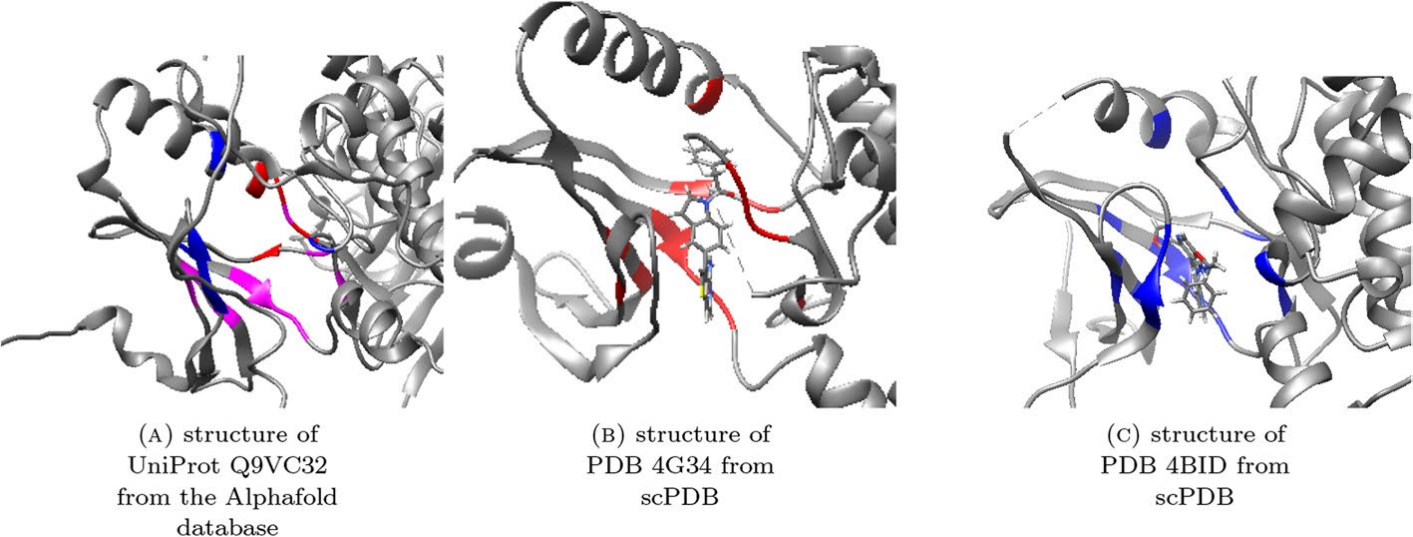
These figures illustrate how our homology-based augmentation assigns residue labels in augmented proteins for a positive binding site candidate. **A** illustrates UniProt protein Q9VC32 as an augmented protein. The red and purple residues correspond to the red residues in (**B**) of the PDB protein 4G34, which are the ligand-binding residues. Similarly, the blue and purple residues correspond to the blue residues in figure (**C**) of the PDB protein 4BID, which are the ligand-binding residues. The purple residues, which form the intersection, attain labels 1.0, while the other colored residues attain labels 0.5. This means that our augmentation method regards the purple residues as the most likely ligand-binding ones

**Table 1:** Residue names for colored sets in Fig. 4

Protein	Color	Label	Chain	Residues	Correspondence in Q9VC32
Q9VC32	Red	0.5	A	PHE156, TYR167, GLY247, PHE249	–
Q9VC32	Purple	1.0	A	ALA119, VAL132, LYS134,	–
				VAL165, MET181, GLU182,	
				TYR183, ALA184, PHE237, ASP248	
Q9VC32	Blue	0.5	A	GLY112, GLN113, GLY114,	–
				GLU152, ILE179, ALA234	
4G34	Red	-	A	VAL606, ALA619, LYS621,	ALA119, VAL132, LYS134,
				LEU642, VAL651, TYR653,	PHE156, VAL165, TYR167,
				MET887, GLN888, LEU889, CYS890,	MET181, GLU182, TYR183, ALA184,
				PHE943, GLY953, ASP954, PHE955	PHE237, GLY247, ASP248, PHE249
4BID	Blue	–	B	GLY687, LYS688, GLY689, VAL694,	GLY112, GLN113, GLY114, ALA119,
				ALA707, LYS709, GLU725, VAL738,	VAL132, LYS134, GLU152, VAL165,
				ILE752, MET754, GLU755, GLN756,	ILE179, MET181, GLU182, TYR183,
				VAL757, ASP807, LEU810, ASP822	ALA184, ALA234, PHE237, ASP248

We use *homology-based augmentation*, which is a form of semi-supervised learning that aims to improve the training by utilizing the large database of protein structures whose binding sites are unlabelled. It is distinguished from the conventional augmentation methods in that it does not rely on transformations applied to the samples during the training. Instead, it pre-computes appropriate “augmented samples” out of the unlabelled database and uses the augmented dataset consisting of the augmented samples during training. Essentially, the augmented samples are selected based on the sequence alignments computed against the proteins in the original training set. In the following, we describe the augmentation method in detail, clarifying its inputs, outputs, procedures, and underlying rationale.

The augmentation method requires a *seed* database $\mathcal {S}^*$ of multi-chain protein-ligand complexes and a *target* database $\mathcal {T}$ of single-chain protein structures. In our instantiation, $\mathcal {S}^*$ was the training set of the scPDB dataset [11], different for each cross-validation fold. For $\mathcal {T}$, we used version 2 of the alphafold protein structure database [38] which contained 992,316 protein structures from the proteome of humans and 47 other key organisms, as well as the Swiss-Prot entries.

The augmentation procedure generates two types of information, collectively constituting the augmented dataset, which is subsequently utilized during training as outlined in "The homology-based augmentation" section. The first type of information denotes the centers of the binding site candidates in proteins in a selected subset of $\mathcal {T}$, labeled either *positive* or *negative*. This is used to augment the BSD training dataset. The second type of information denotes, for each previous positive binding site candidate, the likelihood of each nearby protein residue being a ligand-binding residue. This is used to augment the BRI training dataset.

In the following, we describe the steps of the procedure. The *italicized words* are general terms whose specification may vary depending on one’s needs. Whenever there is an *italicized word*, we provide specific details at the end of the step.

1. In each holo structure of $\mathcal {S}^*$, find ligands *associated to* exactly one chain. As a result, obtain a database $\mathcal {S}$ of protein chains associated to at least one such single-chain ligands. (A chain can be associated to multiple single-chain ligands) We define that a chain and a ligand are *associated to* each other if they have heavy atoms within $4\mathop {\textrm{A}}\limits ^{\circ }$ to each other.
2. Run a *homology search algorithm* with $\mathcal {S}$ as the query database and $\mathcal {T}$ (the database of single-chain protein structures) as the target database. Based on the results, obtain an MSA for each chain in $\mathcal {S}$. For the *homology search algorithm*, we use the software HHBlits [30] with its default setting.
3. For each triplet (*x*, *l*, *y*), composed of:
  1. a query chain *x* in $\mathcal {S}$
  2. a ligand *l* associated to *x* found in step 1 of the procedure and
  3. a target chain *y* aligned with *x* in the MSA,
  determine whether the ligand *l*’s binding site in *x* is *preserved* in *y*. The triplets for which the previous determination was affirmative will be called *preserving*. We define a triplet (*x*, *l*, *y*) as *preserving* if at least half of the residues of *x* that are in close contact with *l* (heavy atoms within $4\mathop {\textrm{A}}\limits ^{\circ }$) are aligned with a residue of *y* in the MSA.
4. For each preserving triplet (*x*, *l*, *y*), find a *proxy center* of the binding site in *y* that corresponds to the ligand *l*’s binding site in *x*. We define the *proxy center* to be the mean of the alpha carbon coordinates of the residues of *y* aligned in the MSA with a residue of *x* in close contact with *l*.
5. On each chain *y* in $\mathcal {T}$ that is involved in at least one preserving triplet, run Fpocket to get an initial list of binding site center candidates. Label a candidate center “positive” if it is within a *lower threshold* from a proxy center obtained in the previous step. Label it “negative” if it is further than a *higher threshold* from any such proxy center. If a candidate center does not fall into these categories, ignore it and exclude it from the dataset. We define the *lower threshold* to be $7.5\mathop {\textrm{A}}\limits ^{\circ }$ and the *upper threshold* to be $30\mathop {\textrm{A}}\limits ^{\circ }$ (justifications of these values are provided in Section 5 of Supplementary Information). Figure 3 illustrates this step using schematic figures.
6. For each positively labeled binding site candidate from the previous step, label residues of *y* with the estimated likelihood of comprising the binding site. The estimate is obtained as a result of “voting” of the homologous chains in $\mathcal {S}$ that gave rise to the binding site. More specifically, among the preserving triplets (*x*, *l*, *y*) whose proxy center gave rise to the binding site (in the sense of the step (5–1)), the proportion of such triplets for which the residue at hand corresponds (in MSA) to a residue in the binding site of *l* is computed. Figure 4 illustrates this step using an actual example.

The assignments of different labels in the previous procedure are based on the following hypotheses:

- The positive binding site label: if a pocket-like site (discovered by a geometry-based BSP method) is surrounded by the sequence fragments that are homologous to the binding site sequence fragments of other proteins, then it is likely to be a binding site.
- The negative binding site label: Even if a site exhibits pocket-like characteristics, it is unlikely to be a binding site if it is distant from any sequence fragments that share homology with the binding site sequence fragments of other proteins in a given seed database.
- The residue labels: Whether a residue near a binding site is considered as a part of the binding site or not can be determined by assessing whether the same holds true for corresponding residues in homologous binding sites.

Note that similar hypotheses have been the basis of template-based methods introduced in "Template-based methods".

Also, it is important to note that we are not arguing that our augmentation procedure always reliably assigns precise labels to unlabelled proteins. Rather, we believe that it is a good approximation that expands on well-founded principles underlying template-based methods and has proven its empirical benefits through our experiments.

### How these approaches address the challenges posed by the existing methods.

#### The problem of CNN-only architectures

Since an attention layer can emulate arbitrarily distant interaction by a single step of operation, it can obviate the problem of *long-term dependency* by keeping the layers relatively shallow while being able to capture global patterns.

#### The problem of excessive post-processing

Given that the fundamental unit of computation in our model architecture is the pocket and residue, its outputs directly align with the resolutions of interest. Therefore, it does not require additional post-processing that might be sub-optimal for the sub-tasks.

#### The problem of under-utilization of existing data sources

Our method addresses this issue through two approaches. Firstly, inter-resolution transfer learning resolves the issue of overlooking more fine-grained information, a limitation observed in certain previous methods for Binding Site Detection (BSD). Secondly, homology-based augmentation offers a mechanism to leverage databases of protein structures whose binding sites are unlabelled, which were previously overlooked by existing works.

### Additional details of training

#### Training the BSD module

To implement the transfer learning described in "The inter-resolution transfer learning" section, the BSD training consists of two stages. The first stage is pre-training the part of the BSD module’s architecture shared by the BRI module, as depicted in Fig. 2. In this process, we append the unshared portion of the BRI module architecture on top of the shared part in the BSD module and then train the combined model for the BRI task. The second stage is fine-tuning the entire original BSD module for the BSD task. In the second stage, to facilitate seamless transfer learning, we freeze the parameters of the parts trained in the first stage up to certain steps of gradient descent.

In both stages, we use a balanced sampling of binding site candidates of positive and negative labels. This is because, among the binding site candidates predicted by Fpocket (on average 33 per protein in scPDB), only a few are actual binding sites, typically only one. Training without such balanced sampling may lead to the model being biased toward the outnumbered label. [17]

In addition, we resolve the similar problem of unbalanced residue labels in the first stage using a weighted loss function. This loss function consists of a weighted sum of terms computed from different residues, where the binding and non-binding residues attain the following weights:

3.7 $$\begin{aligned} w_{pos}=\frac{1}{2n_{pos}},\quad w_{neg}=\frac{1}{2n_{neg}} \end{aligned}$$

where $n_{pos}$ and $n_{neg}$ are the numbers of binding and non-binding residues, respectively.

#### Training the BRI module

To train the BRI module, we exclusively use the positive binding site candidates, which is identical to the procedure used in Deeppocket. This is because, in our intended usage, the BRI module operates on the binding sites detected by the BSD module. Note that this intention is reflected in the evaluation metric *average IOU of binding residues against the closest ligands* as well. All the settings of the first stage of BSD training were maintained, except the balanced sampling of the binding site candidates.

#### The homology-based augmentation

In all stages of training, applying the homology-based augmentation involves adding an auxiliary loss to the original loss (originating from the original dataset). This auxiliary loss is calculated in the same manner but stems from the augmented dataset.

### Datasets

We used scPDB v.2017 [11] as the main dataset for training and validation. In addition, we used three other datasets for the tests: COACH420 [19], HOLO4K [19], and CHEN [9]. To be more specific, we used the training subset of the scPDB dataset provided by [25] for 5-fold cross-validation. This subset excludes proteins with a sequence similarity higher than 90% to the proteins in one of the external test datasets. We used the remaining part of the scPDB as a test dataset. Thus, the test datasets were comprised of the scPDB test set and the external test datasets — COACH420, HOLO4k, and CHEN. The CHEN dataset had holo and apo subsets. Thus, in the tests using the apo subset, we obtained the ground-truth binding sites from the structural alignments with the corresponding holo structures. More specifically, the ligands of the holo structures were superimposed onto the apo structures according to the structural alignments. The characteristics of each test dataset and the details of the structural alignments are described in Section 2 of Supplementary Information. For instance, the basic properties of each dataset such as the number of proteins and the average number of binding sites are presented in Table 1 of Supplementary Information.

### Evaluation methods

We use three evaluation metrics: (1) *the success rate for detection* (success rate) (2) *the average IOU of binding residues against the closest ligands* (IOU) (3) *the average IOU of the binding residues against the successfully detected ligands* (conditional IOU). The metrics evaluate different combinations of BSD and BRI performances. The success rate metric evaluates BSD performance, the IOU metric evaluates BRI performance but it is also influenced by BSD performance, and the conditional IOU metric aims to evaluate BRI performance alone. We give additional details with regard to how these evaluation metrics compare to their counterpart metrics introduced in the previous literature in Supplementary Information.

To provide a formal definition of each metric, we shall adopt the following notations:

- $n^{(i)}$ is the number of ground-truth ligands bound to the *i*-th protein.
- $\left\{ l^{(i)}_{1},\cdots , l^{(i)}_{n^{(i)}}\right\}$ is the set of ground-truth ligands bound to the *i*-th protein.
- $\left\{ (c^{(i)}_1,BR^{(i)}_1)\cdots , (c^{(i)}_{n^{(i)}}, BR^{(i)}_{n^{(i)}})\right\}$ is the set of predictions of the method to evaluate.
- $\left\{ TBR^{(i)}_1,\cdots ,TBR^{(i)}_{n^{(i)}}\right\}$ is the set of *true binding residue indices*, where $TBR^{(i)}_j$ is defined to be the set of residues in the *i*-th protein that is within $4\mathop {\textrm{A}}\limits ^{\circ }$ from $l^{(i)}_j$

The success rate metric measures the correspondence between the predicted binding site centers $\left\{ c^{(i)}_1,\cdots ,c^{(i)}_{n^{(i)}}\right\}$ and the positions of the ground-truth ligands $\left\{ l^{(i)}_1,\cdots ,l^{(i)}_{n^{(i)}}\right\}$. For each *i*, we compute the F1 score (the harmonic mean of precision and recall) based on the definition of *detection*. Specifically, we define that $c^{(i)}_j$ is a *correct detection* of $l^{(i)}_k$ when $c^{(i)}_j$ is within $4\mathop {\textrm{A}}\limits ^{\circ }$ (a threshold commonly used in the literature e.g. [1] and [25] from any ligand of $l^{(i)}_k$. In other words, we define that detection is correctly performed when the Distance from Center to Atom (DCA) is $<4\mathop {\textrm{A}}\limits ^{\circ }$. Then, the F1 scores are weighted-averaged (weighted by $n^{(i)}$) over the proteins. In summary, we obtain this metric as

3.8 $$\begin{aligned} \left( \sum _{i}n^{(i)}\cdot \frac{2}{\frac{1}{P^{(i)}}+\frac{1}{R^{(i)}}} \right) \bigg / \left( \sum _{i}n^{(i)} \right) \end{aligned},$$

where $P^{(i)}$ is the *precision* defined as follows:

3.9 $$\begin{aligned} P^{(i)} = \frac{\#\left\{ 1\le j\le n^{(i)}:c^{(i)}_j\text { detects one of }l^{(i)}_1,\cdots l^{(i)}_{n^{(i)}}\right\} }{n^{(i)}} \end{aligned}$$

and $R^{(i)}$ is the *recall* defined as follows:

3.10 $$\begin{aligned} R^{(i)} = \frac{\#\left\{ 1\le k\le n^{(i)}:l^{(i)}_k\text { is detected by one of }c^{(i)}_1,\cdots ,c^{(i)}_{n^{(i)}}\right\} }{ n^{(i)}} \end{aligned}$$

This is a BSD metric since it involves only the predicted binding site centers, not the predicted binding residues.

The IOU metric compares the predicted binding residues $BR^{(i)}_j$ with the true binding residues $TBR^{(i)}_{\phi ^{(i)}(j)}$ of the ligand $l^{(i)}_{\phi ^{(i)}(j)}$ closest to the predicted binding site center $c^{(i)}_j$. Here, the index $\phi ^{(i)}(j)$ of the closest ligand is defined as

3.11 $$\begin{aligned} \phi ^{(i)}(j) = \mathop {\textrm{argmin}}\limits _{k=1}^{n^{(i)}}DCA(c^{(i)}_j, l^{(i)}_k) \end{aligned}$$

The comparison is performed in terms of intersection over union (IOU), and the quantity is averaged over all pairs of (*i*, *j*). In summary, we obtain the second metric as

3.12 $$\begin{aligned} \left( \sum _{i}\sum _{j=1}^{n^{(i)}}\frac{\#(BR^{(i)}_j\cap TBR^{(i)}_{\phi ^{(i)}(j)})}{\#(BR^{(i)}\cup TBR^{(i)}_{\phi ^{(i)}(j)})}\right) \bigg / \left( \sum _{i}n_i\right) \end{aligned}$$

Although this is essentially a BRI metric, it also depends on BSD performance due to how the $\phi ^{(i)}(j)$ is defined. In particular, if the predicted center $c^{(i)}(j)$ is far from any ligand, the set of predicted binding residues ${\hat{R}}_j^{(i)}$ does not contribute to the metric.

The conditional IOU metric is similar to the IOU metric, but it aims to eliminate the previously mentioned problem of the IOU metric being dependent on BSD performance. It does so by focusing on the case that the predicted binding sites are close to at least one ligand. In summary, we obtain the metric as

3.13 $$\begin{aligned} \left( \sum _{i}\sum _{j\in S^{(i)}}\frac{\#(BR^{(i)}_j\cap TBR^{(i)}_{\phi ^{(i)}(j)})}{\#(BR^{(i)}\cup TBR^{(i)}_{\phi ^{(i)}(j)})}\right) \bigg / \left( \sum _{i}\# S^{(i)} \right) \end{aligned}$$

where

3.14 $$\begin{aligned} S^{(i)} = \left\{ j=1,\cdots ,n^{(i)}:DCA(c^{(i)}_j,l^{(i)}_{\phi ^{(i)}(j)})<4\mathop {\textrm{A}}\limits ^{\circ }\right\} \end{aligned}$$

### Baseline methods

We compared our method to the previous state-of-the-art deep learning methods, which are based on CNN: Deeppocket [1], Kalasanty [35] and DeepSurf [25]. All these methods are briefly explained in "Structure-based deep learning methods" section. For Deeppocket and DeepSurf, we have trained their parameters from scratch according to our dataset splits. However, for Kalasanty, we used the parameters released by the authors due to the high computational costs of training. It is important to note that the parameters of Kalasanty were trained on the entire scPDB v.2017 dataset. To be specific, the training data of Kalasanty may have included data whose protein sequences are similar (similarity above 90%) to those in the test dataset. Thus, the Kalasanty method has an advantage in terms of the coverage of the training dataset compared to the other methods when they are evaluated on the external test datasets.

The definitions of ${\hat{c}}_1,\cdots , {\hat{c}}_n$ for the baseline methods are mostly natural and derived directly from the original papers. All methods produce a ranked list of predictions; we limit them to produce only the top-*n* outputs. Also, they compute the centers of predicted binding sites in their evaluation, so we can compute ${\hat{c}}_i$ as prescribed.

However, not all baseline models output the predicted binding sites at the residue level. Thus, it is necessary to map their outputs to sets of residues ${\hat{R}}_1,\cdots , {\hat{R}}_n$. For example, in Deeppocket [1], the authors used the distance threshold $2.5\mathop {\textrm{A}}\limits ^{\circ }$ (performed best in their validation set) to determine the binding residues based on the segmented voxels; therefore, we followed the same procedure. For Kalasanty [35] and DeepSurf [25], the authors introduced a method to convert their predictions to atom-level predictions (which was implemented in their code); therefore, we regarded the residues containing at least one such predicted binding atom as the predicted binding residues.

### Ablation study

We conducted an ablation study to assess the effectiveness of each component of our proposed method. We considered the omission of the following components:

- The use of local features extracted by the CNN
- The inter-resolution transfer learning
- The homology-based augmentation

In the ablation of “the use of local features extracted by the CNN”, we removed the CNN component from our model. To be more specific, the hidden vectors for the attention layers were directly obtained from the one-hot encoding layer for the amino acid types, followed by the token embedding layer. To compensate for the loss of model complexity, we added two more attention layers to the default configuration of BRI and BSD model architecture.

## Results

**Table 2:** (BSD metric) F1 success rate for detection

	scPDB(held-out)	COACH420	HOLO4k	CHEN-holo	CHEN-apo
DeepSurf	$62.4\pm 1.3$	$43.6\pm 1.3$	$59.7\pm 1.4$	$24.5\pm 1.4$	$22.3\pm 1.2$
Kalasanty	$70.0\pm 0.0$	$50.8\pm 0.0$	$44.9\pm 0.0$	$28.5\pm 0.0$	$27.1\pm 0.0$
DeepPocket	$67.9\pm 0.4$	$55.7\pm 0.7$	$72.2\pm 0.2$	$\varvec{42.4\pm 0.3}$	$34.5\pm 1.2$
Ours	$\varvec{70.1\pm 0.4}$	$\varvec{59.1\pm 0.3}$	$\varvec{77.0\pm 0.6}$	$41.2\pm 1.2$	$36.5\pm 0.2$
Ours(no CNN)	$69.2\pm 0.7$	$57.7\pm 0.3$	$75.1\pm 0.2$	$41.7\pm 1.0$	$\varvec{36.7\pm 1.0}$
Ours(no transfer)	$61.9\pm 0.3$	$53.9\pm 0.3$	$70.0\pm 0.5$	$39.8\pm 0.5$	$35.1\pm 0.6$
Ours(no homology)	$68.3\pm 1.1$	$57.3\pm 0.9$	$75.1\pm 1.1$	$39.9\pm 0.5$	$34.6\pm 0.6$

The mean and standard deviation are calculated based on the metric values for five different cross-validation folds

The bold text indicates the models that performed best on the corresponding datasets

**Table 3:** (BSD + BRI metric) average IOU of binding residues against the closest ligands

	scPDB(held-out)	COACH420	HOLO4k	CHEN-holo	CHEN-apo
DeepSurf	$0.288\pm 0.007$	$0.194\pm 0.006$	$0.207\pm 0.005$	$0.104\pm 0.003$	$0.085\pm 0.005$
Kalasanty	$0.260\pm 0.000$	$0.183\pm 0.000$	$0.146\pm 0.000$	$0.101\pm 0.000$	$0.092\pm 0.000$
Deeppocket	$0.440\pm 0.002$	$0.313\pm 0.003$	$0.277\pm 0.03$	$0.190\pm 0.005$	$0.186\pm 0.003$
Ours	$\varvec{0.490\pm 0.003}$	$\varvec{0.398\pm 0.004}$	$\varvec{0.346\pm 0.002}$	$\varvec{0.287\pm 0.004}$	$\varvec{0.264\pm 0.004}$
Ours (no CNN)	$0.467\pm 0.003$	$0.357\pm 0.005$	$0.315\pm 0.002$	$0.247\pm 0.005$	$0.227\pm 0.005$
Ours (no homology)	$0.473\pm 0.003$	$0.387\pm 0.007$	$0.341\pm 0.003$	$0.285\pm 0.010$	$0.257\pm 0.004$

The mean and standard deviation are calculated based on the metric values for five different cross-validation folds

The bold text indicates the models that performed best on the corresponding datasets

**Table 4:** (BRI metric) average IOU of binding residues against the detected ligands

	scPDB(held-out)	COACH420	HOLO4k	CHEN-holo	CHEN-apo
DeepSurf	$0.402\pm 0.010$	$0.419\pm 0.013$	$0.330\pm 0.007$	$0.372\pm 0.019$	$0.336\pm 0.020$
Kalasanty	$0.356\pm 0.000$	$0.362\pm 0.000$	$0.344\pm 0.000$	$0.333\pm 0.000$	$0.323\pm 0.000$
Deeppocket	$0.595\pm 0.002$	$0.506\pm 0.005$	$0.371\pm 0.004$	$0.395\pm 0.008$	$0.382\pm 0.006$
Ours	$\varvec{0.643\pm 0.004}$	$\varvec{0.585\pm 0.007}$	$\varvec{0.415\pm 0.002}$	$\varvec{0.495\pm 0.010}$	$\varvec{0.473\pm 0.004}$
Ours(no CNN)	$0.624\pm 0.002$	$0.548\pm 0.004$	$0.395\pm 0.002$	$0.450\pm 0.014$	$0.419\pm 0.006$
Ours(no homology)	$0.628\pm 0.003$	$0.567\pm 0.009$	$0.412\pm 0.002$	$0.481\pm 0.007$	$0.449\pm 0.010$

The mean and standard deviation are calculated based on the metric values for five different cross-validation folds

The bold text indicates the models that performed best on the corresponding datasets

**Fig. 5:**
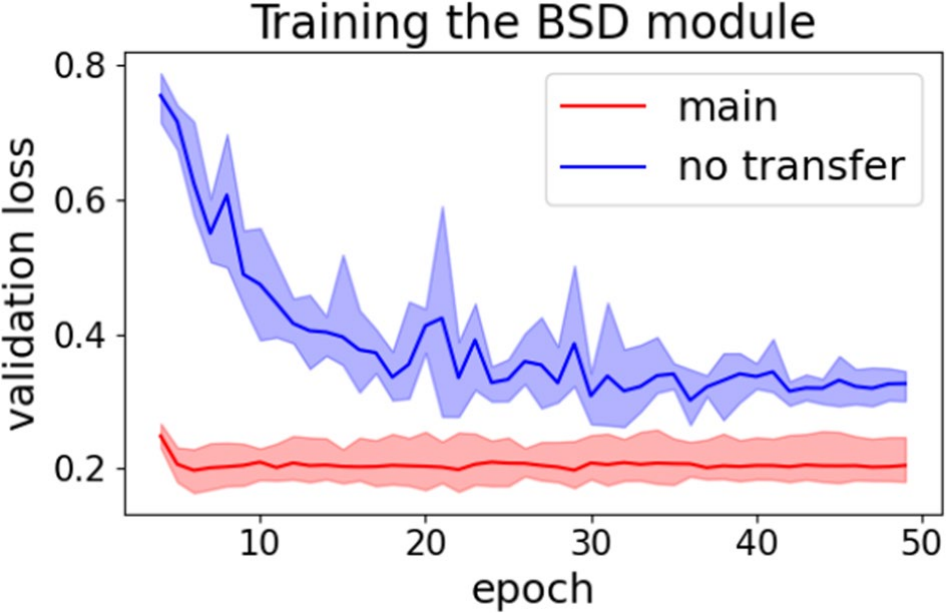
The effect of transfer learning on BSD training

**Fig. 6:**
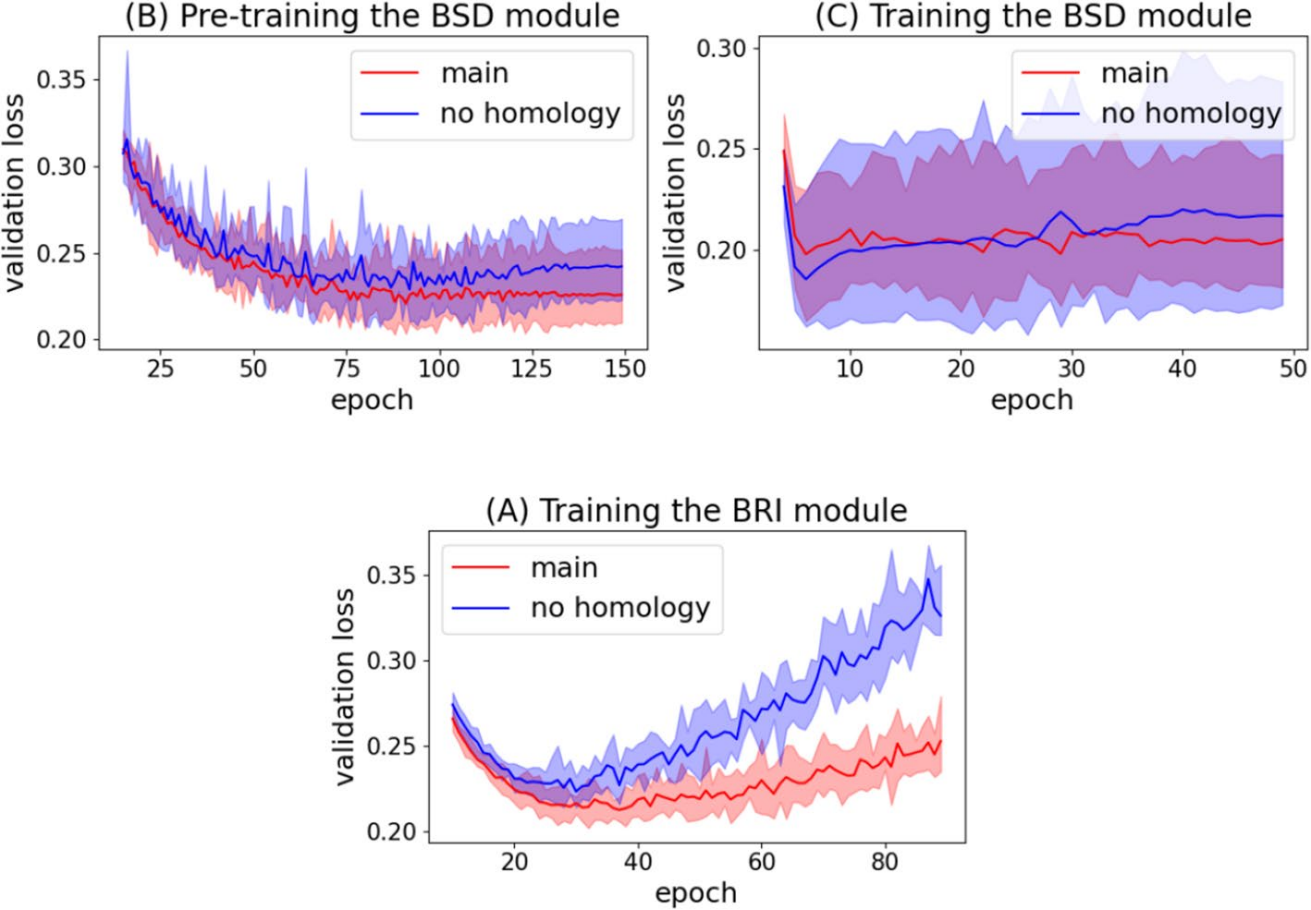
The effect of homology-based augmentation on training

The experiment results are summarized in Tables 2, 3, 4, Figs. 5, and 6.

### Performance enhancements

Tables 2, 3, and 4 show that our method significantly outperforms the baseline methods in both BSD and BRI. The average performance over datasets are improved by $4.1\%$ (BSD), $27.0\%$ (BSD and BRI combined), and $16.1\%$ (BRI) compared to the next best method. Although there is an exception where Deeppocket outperforms our method in BSD on CHEN-holo, the performance gap ($1.2\%p$) is relatively insignificant compared to the average improvement ($3.1\%p$) across other datasets.

### Ablation results

The ablation results show that the use of CNN as a local feature extractor, the inter-resolution transfer learning, and the homology-based augmentation are all effective. With the omission of each component, the average performance over datasets dropped by the following amounts:

- The use of CNN: $1.2\%$ (BSD), $9.6\%$ (BSD + BRI), $6.7\%$ (BRI)
- The inter-resolution transfer learning: $8.2\%$ (BSD)
- The homology-based augmentation: $3.1\%$ (BSD), $2.4\%$ (BSD + BRI), $2.8\%$ (BRI)

### The effects of additional data sources

Figures 5 and 6 show the effects of our methods for leveraging additional data sources in more detail. Figure 6 shows that the homology-based augmentation method alleviated over-fitting during all stages of training. Figure 5 shows that the inter-resolution transfer learning significantly accelerated convergence and improved loss.

## A case study

We conducted a case study to demonstrate our model’s applicability in real-world drug discovery scenarios. We chose Human Serum Albumin (HSA) as the target protein due to its relevance to drug discovery and its ability to bind to various molecules at different sites [8, 28, 29]. To examine our model’s performance on HSA, we referenced two prior studies on the binding sites of HSA [8, 40], and analyzed the compatibility of our BSD and BRI modules’ predictions with the findings of the studies.

The remaining parts of this section are organized as follows:

- In "The structure and binding sites of HSA" section, we briefly explain the structure of HSA, and elucidate what the reference papers [8, 40] revealed about its binding sites.
- In "Basic settings" section, we explain the basic settings of our case study experiments.
- In :BSD" section, we analyze our BSD module’s performance on HSA, based on the findings of [8].
- In "BRI" section, we analyze our BRI module’s performance on HSA, based on the findings of [40].

**Fig. 7:**
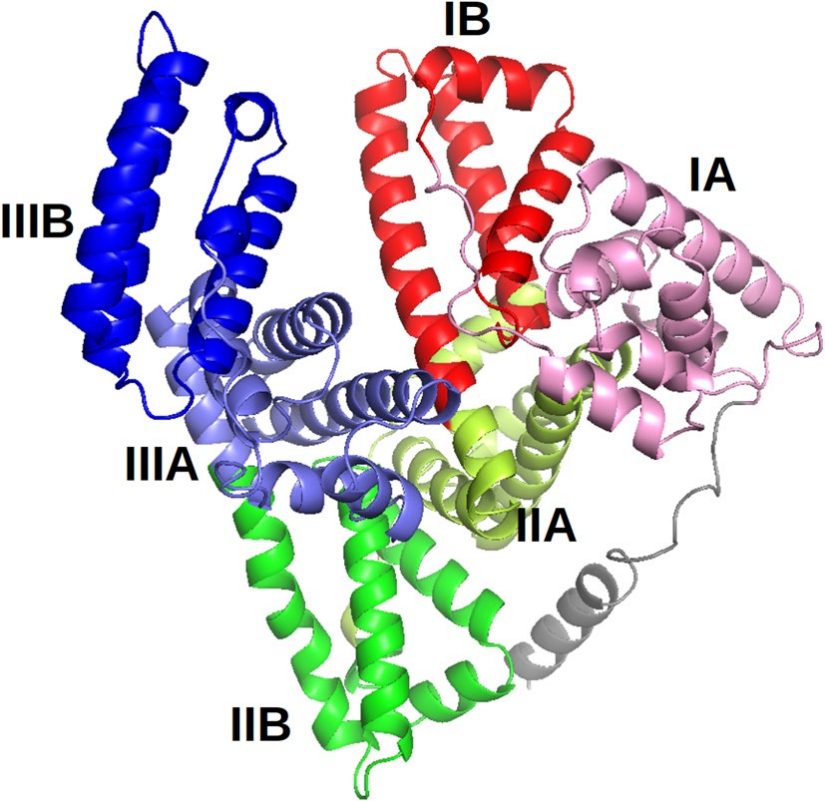
The subdomains of HSA

### The structure and binding sites of HSA

HSA is composed of three homologous domains (I, II, III), each composed of two subdomains A and B, as depicted in Fig. 7 [8, 29]. The authors of [8] performed a large-scale survey of HSA binding sites based on 142 crystal structures involving HSA. By analyzing the complexes, they identified 14 different binding sites as well as their frequencies. They noted that binding sites IB, IIA and IIIA occurred the most frequently. Inspired by this result, the authors of [40] provided a more detailed analysis of the binding sites at the IB subdomain. They did so by inspecting the crystal structures of six oncology drugs (9-amino-camptothecin, camptothecin, idarubicin, teniposide, etoposide, and bicalutamide) each forming complex with HSA. In particular, for each structure, they identified all the key residues of HSA and their interaction types with the drug molecule.

### Basic settings

We designed the analyses in a way that allowed us to faithfully assess our model’s real-world applicability.

Firstly, before the analyses, we trained our model with a new dataset split (different from the ones used in our main experiments) to prevent data leakage. Specifically, we removed all 42 “albumin” structures from the scPDB v.2017 dataset, randomly sampled a validation set of size 1000 from the remaining structures, and took all the other proteins as the training set. Moreover, we ensured that there was no leakage from the homology-based augmentation by using a new augmentation dataset generated from the new training set.

Secondly, our model’s predictions are based on the HSA structure input provided by the Alphafold database [38], structure ID: AF-P02768-F1). Therefore, achieving a good performance under this setting would imply that our model can be used to predict the binding sites of a protein without any known experimental structures. In particular, one can make use of the publicly available Alphafold database in the predictions.

### BSD

#### Experiment procedure

First, we obtained our model and Deeppocket’s BSD module’s predictions on 15 binding sites identified by [8]. This means that the predictions were made based on 15 different inputs, where we set the “binding site center” to be the mean of alpha carbon coordinates of residues comprising one of the binding sites. The indices of residues comprising each binding site were provided by [8]. Note that we re-trained the Deeppocket model with a new dataset split to avoid data leakage, following the same approach we used for our model.

Then, we assessed each model’s predictions by comparing them with the ranks of the frequencies of the binding sites as recorded in [8].

#### Results and analysis

**Fig. 8:**
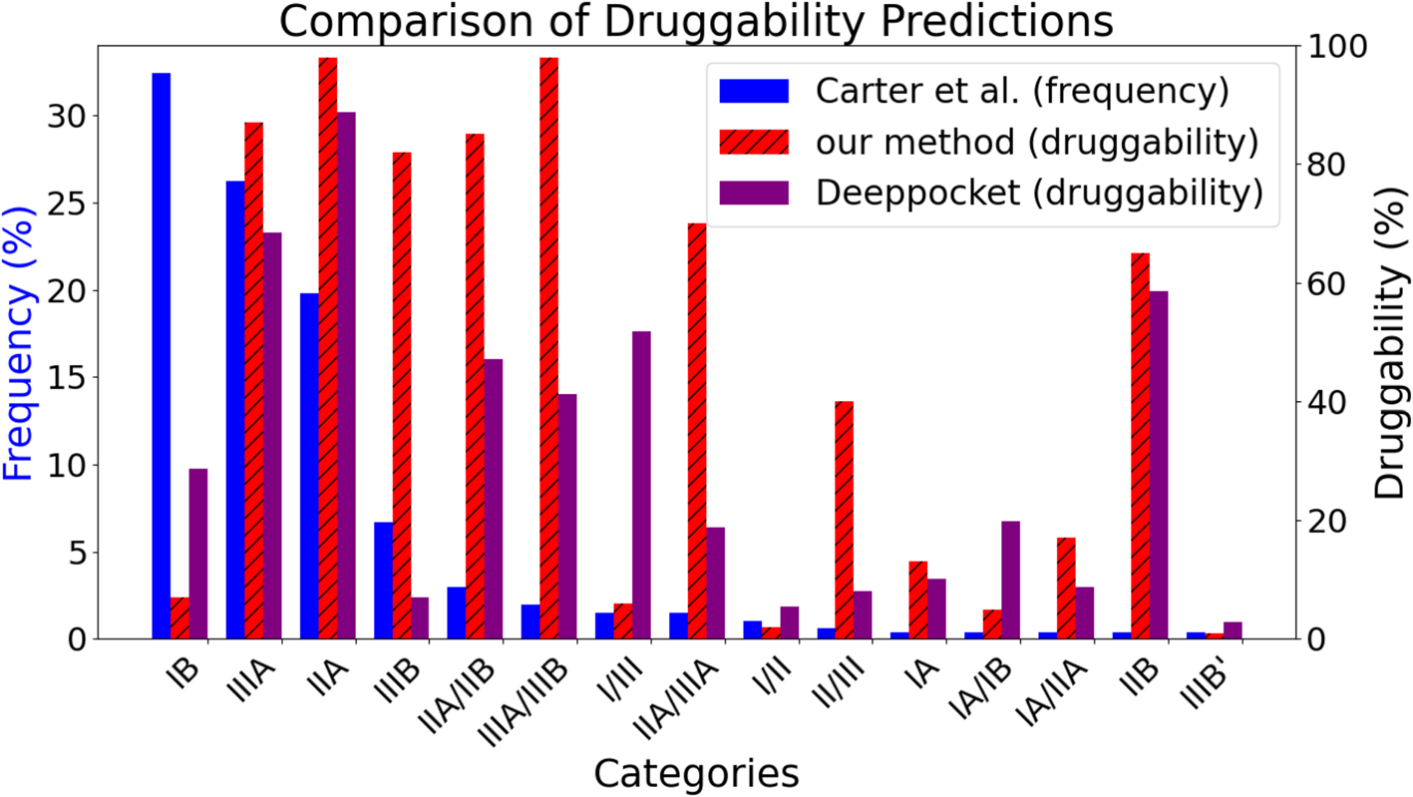
A comparison between our BSD module’s predictions and the frequencies from [8]

The results are summarized in Fig. 8. Our model successfully assigned high (more than $70\%$) druggability scores on the second to sixth most frequent binding sites. Moreover, these five binding sites were those that scored the highest. The probability that the five binding sites that scored the highest by a random prediction are among the six most frequent ones is only $0.2\%$. This shows the statistical significance of our model’s ability to replicate binding sites’ frequency ranks. On the other hand, although Deeppocket assigned high druggability scores on the second and third most frequent binding sites, it failed to do so on the fourth to sixth most *frequent* ones.

### BRI

#### Experiment procedure

First, we predicted the binding site residues in the IB subdomain of HSA using our model and all the baseline methods—Deeppocket, Kalsanty and, DeepSurf. When obtaining our model’s (resp. Deeppocket’s) predictions, we set the “binding site center” as the mean of alpha carbon coordinates of the IB subdomain residues, and ran the BRI module (resp. the *segmentation model*). Note that we re-trained the Deeppocket and DeepSurf models with a new dataset split to avoid data leakage, just as we did for our model.

Then, we visually inspected the binding residues predicted by different methods, comparing them with the ground truth determined based on the six molecules from [40]. We defined the ground truth as the set of residues in the Alphafold-predicted structure that correspond to binding residues found in one of the six drug-bound structures from [40] via the atom distance ($4\mathop {\textrm{A}}\limits ^{\circ }$) criteria used throughout this study.

#### Results and analysis

**Fig. 9:**
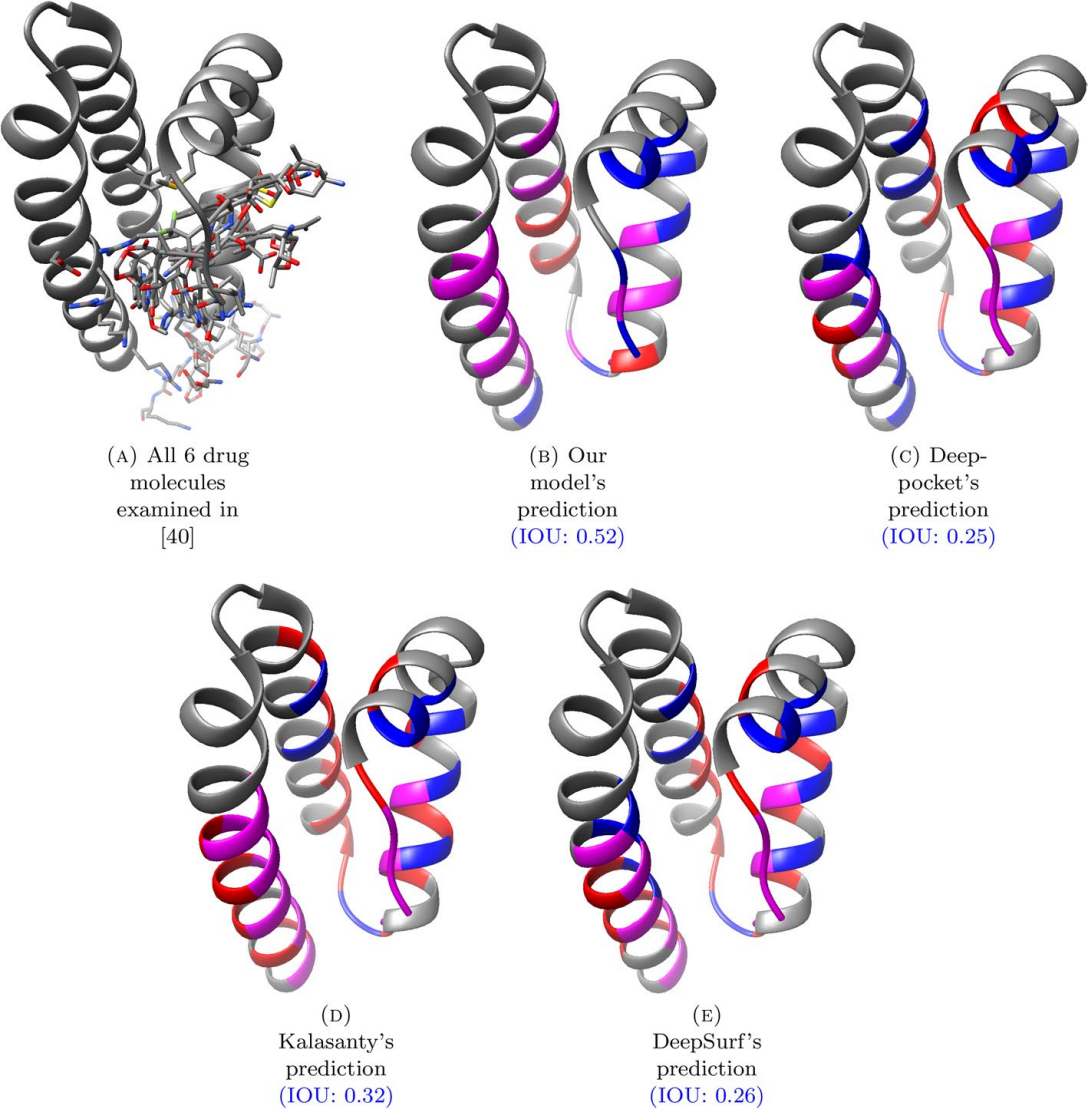
These figures illustrate the binding residue prediction results for the IB subdomain of HSA (based on the Alphafold-predicted structure AF-P02768-F1). The ground truth residues are determined based on the 6 drug molecules examined in [40]. The purple, red and blue residues indicate the true-positive, false-positive and false-negative binding site residues respectively. Therefore, the larger the purple region is compared to the regions with the other colors, the better the prediction is

The results are summarized in Fig. 9 and Table 5. From the figures and the IOU metrics, it is clear that our model’s prediction best matched the ground truth. In particular, while the baseline models tend to alternate (in the sequence order) between accurate and inaccurate predictions in the regions where they are accurate, our model’s predictions are “clean” in the sense that there is no such alternation. This can be partly due to the use of residue-level computations that obviate the need for proximity-based post-processing, which is an intended benefit.

**Table 5:** Residue Names for the predictions in Fig. 9

Method	Residues
Ground truth	VAL140, ARG141, PRO142, VAL146, MET147, ALA150, PHE158, LYS161, TYR162, GLU165, ILE166, HIS170,
	TYR172, PHE173, TYR185, PHE189, LEU206, ASP207, LEU209, ARG210, GLY213, LYS214, SER217, ARG221
Ours	**ARG141**, **TYR162**, **GLU165**, **ILE166**, ARG169, **HIS170**, **PHE173**, GLU177, LEU178, PHE181,
	ALA182, **TYR185**, **PHE189**, **LEU206**, **ASP207**, **LEU209**, **ARG210**, **GLY213**, **LYS214**, **SER217**
Deeppocket	**VAL140**, **ARG141**, **PRO142**, GLU143, CYS148, PHE151, LEU159, **TYR162**, LEU163, **ILE166**,
	ALA167, **HIS170**, PRO171, TYR174, ALA182, LYS186, **ASP207**, **ARG210**, ASP211, **LYS214**, ALA215
Kalasanty	**VAL140**, **ARG141**, **PRO142**, GLU143, CYS148, LEU159, **TYR162**, LEU163, TYR164, **ILE166**, ALA167,
	**HIS170**, PRO171, TYR174, ALA175, LEU178, LEU179, ALA182, LYS186, THR190, **LEU206**, **ASP207**, GLU208,
	**LEU209**, **ARG210**, ASP211, GLU212, **GLY213**, **LYS214**, ALA215, **SER217**, ALA218, GLN220, **ARG221**
DeepSurf	**VAL140**, **ARG141**, **PRO142**, GLU143, CYS148, LEU159, LYS160, **TYR162**, LEU163,
	**ILE166**, ALA167, **HIS170**, PRO171, TYR174, ALA175, LEU179, ALA182, LYS186, **ASP207**,
	**ARG210**, ASP211, GLU212, **LYS214**, ALA215, SER216, **SER217**, LYS219, GLN220, **ARG221**

The true positive residues are marked in bold text

## Discussion and conclusions

Existing BSP methods had limitations regarding (1) relying solely on CNN architecture, (2) involving excessive post-processing, and (3) under-utilizing existing data sources. We provided a model architecture and training method that resolve all these issues. We showed that the resulting algorithm significantly outperformed the existing methods in the benchmark datasets and a case study.

One problem that the current study did not address was the models’ selectivity for homologous proteins. We conducted a small experiment on this issue, detailed in Section 4 of Supplementary Information, and the results suggest a potential reduction in selectivity due to our homology-based augmentation. Although this did not negatively impact the overall performance in that experiment, properly addressing this issue could lead to further improvements. Future work might focus on this problem.

## Supplementary information

Supplementary file 1.

## Data Availability

The data and instructions for inference (including the source code and a docker image) are available at https://github.com/deargen/bsp_public.
