# Supplementary material for: Turbocharging protein binding site prediction with geometric attention, inter-resolution transfer learning, and homology-based augmentation
Source: BMC Bioinformatics. 2024 Sep 20;25:306. doi: 10.1186/s12859-024-05923-2 (PMC11416008; doi:10.1186/s12859-024-05923-2)
Supplement: Supplementary file 1 [file 12859_2024_5923_MOESM1_ESM.pdf]

SUPPLEMENTARY INFORMATION

1. Additional details of the model and training

1.1. **Grid featurization.** As explained in 3.1.2 and 3.1.3 of the manuscript, our BSD and BRI modules take a sequence of 3D voxelized images as input, where each image represents the local environment of a residue. The process of making such voxelized images, the "grid featurization", is as follows:

(1) Collect coordinates $\mathbf{x}_i \in \mathbb{R}^3$ $(1 \le i \le n)$ of the heavy (non-hydrogen) atoms in the protein.
(2) Collect feature vectors $f_i \in \mathbb{R}^{d_{feature}}$ $(1 \le i \le n)$ of the heavy atoms.
(3) Given a choice of *grid axes* $(\mathbf{e}_1, \mathbf{e}_2, \mathbf{e}_3)$, a cubical grid is laid such that the centers of the voxels in the grid become

$$t + \sum_{i=1}^{3} r(a_i - \frac{L-1}{2})\mathbf{e}_i \quad ((a_1, a_2, a_3) \in \{0, \cdots, L-1\}^3) \tag{1.1}$$

where t is the grid center (the alpha carbon coordinate), r is the *grid resolution* and L is the *grid size.*
(4) Compute feature vectors corresponding to each voxel in the grid, by summing up those of nearby heavy atoms.

The same process was used in [1], [10], and [15], although the grids in those methods are not laid on the protein residues.

In our grid featurization process:

- The grid resolution is 1Å and the grid size is 16.
- The atom features are of dimension 18 and include atom types, hybridization, degree, partial charge, and aromaticity ([15], [1]).

1.2. **Grid alignment.** When constructing the grids, we do not use arbitrary (xyz) axes but rather use axes aligned to the orientations of the residues. (See 1.3) This is analogous to the grid orientation used in [10], which sets the z-axis to the normal vector of the protein surface at the grid center (which is on the protein surface).

1.3. **The residue orientation.** Our model uses a concept of "residue orientation" in the geometric attention layers and the grid alignment process. We define the residue orientation in terms of the relative positions of atoms surrounding the alpha carbon, as in [6]. More precisely, when $\mathbf{x}_1$, $\mathbf{x}_2$ and $\mathbf{x}_3$ are the coordinates of N, $C\alpha$ and C (the carbon that is not $C\beta$ and adjacent to $C\alpha$), the rotation matrix $R = (\mathbf{e}_1 \ \mathbf{e}_2 \ \mathbf{e}_3)$ that we refer to as "orientation" is obtained as follows:

$$\begin{aligned}
\mathbf{v}_1 &= \mathbf{x}_3 - \mathbf{x}_2 \\
\mathbf{v}_2 &= \mathbf{x}_1 - \mathbf{x}_2 \\
\mathbf{e}_1 &= \mathbf{v}_1 / \|\mathbf{v}_1\| \\
\mathbf{u}_2 &= \mathbf{v}_2 - (\mathbf{e}_1 \cdot \mathbf{v}_2)\mathbf{e}_1 \\
\mathbf{e}_2 &= \mathbf{u}_2 / \|\mathbf{u}_2\| \\
\mathbf{e}_3 &= \mathbf{e}_1 \times \mathbf{e}_2
\end{aligned} \tag{1.2}$$

1.4. **Satisfaction of SE(3)-invariance.** A function is *SE(3)-invariant* if its output remains unchanged when SE(3) transformations (translations, rotations or compositions of them) are applied to the input. In our context, when $\{v_i\}_{i=1}^N$ are the coordinates of the protein atoms, $\{f_i\}_{i=1}^N$ are the feature vectors of the protein atoms, and $\{T_i\}_{i=1}^n$ are the local frames related to the protein residues, a function $f(\{v_i\}_{i=1}^N, \{f_i\}_{i=1}^N \{T_i\}_{i=1}^n)$ is SE(3)-invariant if, for any SE(3) transformation T, we have

$$f(\{Tv_i\}_{i=1}^N, \{f_i\}_{i=1}^N, \{TT_i\}_{i=1}^n) = f(\{v_i\}_{i=1}^N, \{f_i\}_{i=1}^N, \{T_i\}_{i=1}^n) \tag{1.3}$$

The SE(3)-invariance is a desired property for the structure-based BSP models. This is because the binding site information should remain unchanged regardless of the reference frame. It is known that in such cases, incorporating the SE(3)-invariance property into the model may enhance the robustness of the predictions ([5]).

Our BSD and BRI modules are SE(3)-invariant. This is mainly due to two facts: the grid alignment process makes the CNN-produced residue features SE(3)-invariant, and our geometric attention mechanism is SE(3)-invariant. Note that the latter can be derived in an almost identical way to the proof presented in [6] (Supplementary Information, page 28).

1.5. **Reference point embedding.** For the BSD module, we additionally use *reference point embedding*, which informs each residue of the location of the center of the binding site candidate. More precisely, when $\mathbf{v}$ is the center, $f(R_i^{-1}(\mathbf{v} - \mathbf{t}_i))$ is added to the hidden vectors in the BSD module right after the parts shared with the BRI module, where f is a learnable linear function. The embedding takes this specific form to retain the SE(3)-invariance of the model.

1.6. **Random perturbation augmentation.** During training, we use *random perturbation augmentation*, which rotates the residue orientations in a small magnitude before inputting them into the attention layers. This is to promote diversity in the geometric information while not completely forgetting the original residue orientation. Note that these rotated residue orientations were computed once for each step of the training and used only in the attention layers but not in the grid featurization.

1.7. **Model hyperparameters.**

1.7.1. *CNN.* We used the same hyperparameters as [10] for the 3D BottleNeck ResNet model:

- Input channel dimension: 18
- Output channel dimensions of the blocks: 64, 128, 256, 512
- Number of ResNet units in the blocks: 2, 2, 2, 2

Our pytorch implementation of the architecture including the hyperparameters can be found in `https://github.com/deargen/bsp_public/blob/main/src/model/bottleneck_resnet.py`.

1.7.2. *Attention.* For individual attention layers, we used the same default hyperparameters as Alphafold:

- hidden dimension: 128
- number of attention heads: 12
- dimension of query and key vectors: 16,
- dimension of value vectors: 16
- number of attention points: 4
- dropout rate: 0.1

At the end of each layer, we used a two-layer point-wise feed-forward network with the intermediate hidden dimension 512.

For the part of the architecture that BSD and BRI modules share, we used three attention layers. For the BSD module, we used one additional attention layer.

1.8. **Pre-training BSD module.**

- Minibatch size: 16
- Batch sampling: balanced BSD labels (See 3.5.1 of the manuscript)
- Loss function: balanced binary cross entropy (See 3.5.1 of the manuscript)
- Reduction of loss terms from individual datapoints: mean
- Peak learning rate: 1e-4
- Learning rate schedule: one-cycle cosine scheduling ([8]) with 6000 warmup steps and 60000 total steps
- Optimizer: AdamW with $\beta_1 = 0.9$, $\beta_2 = 0.999$ and weight decay rate 0.01

1.9. **Fine-tuning BSD module.**

- Minibatch size: 16
- Batch sampling: balanced BSD labels (See 3.5.1 of the manuscript)
- Loss function: binary cross entropy
- Reduction of loss terms from individual datapoints: mean
- Peak learning rate: 1e-6
- Learning rate schedule: one-cycle cosine scheduling ([8]) with 4000 warmup steps and 20000 total steps
- Weight freezing: Up to 8000 steps, the parameters from the part of the architecture shared with the BRI module are frozen (See 3.5.1 of the manuscript)
- Optimizer: AdamW with $\beta_1 = 0.9$, $\beta_2 = 0.999$ and weight decay rate 0.01

1.10. **Training BRI module.** We used the same details as in 1.8, except the balanced batch sampling. When training BRI module, as explained in 3.5.2 of the manuscript, we only used the binding sites with positive BSD labels.

2. Datasets

In our experiments, we used the datasets scPDB, COACH420, HOLO4K, and CHEN. As explained in 3.6 of the manuscript, we used scPDB for a held-out cross validation, and used the other ones as external test datasets.

2.1. **Characteristics of the datasets.** ScPDB ([4]) is a large database of binding sites from the Protein Data Bank. COACH420 ([17]) consists of 420 single-chain structures from the COACH dataset. HOLO4K ([13]) consists of 4009 larger multi-chain structures. The CHEN dataset ([3]) is smaller, but covers a wide range of non-homologous structures. Also, it has 104 holo structures with apo counterparts, where corresponding holo and apo structures have the same SCOP family, 80% sequence similarity, and a TM score above 0.5. These holo and apo structures resulted in "CHEN-holo" and "CHEN-apo" datasets used in our experiments.

2.2. **Ligands used for evaluations.** Evaluation of binding sites depends on what "ligands" are considered to constitute binding sites. "Biologically irrelevant" ligands such as additives are usually excluded in this calculation. Therefore, we will clarify what ligands we considered for each dataset. For scPDB and CHEN, we used the ones the original databases provided. For COACH420 and HOLO4K, we used the ligands in the PDB files provided by the p2rank repository ([7], [11]), which are those with PDB ligand code listed in "biologically relevant ligands" curated by MOAD 2013 database ([2]).

2.3. **Structural alignments of apo structures and holo structures.** To evaluate the BSP algorithms' performances on the CHEN-apo dataset, we projected the ligands in CHEN-holo to the corresponding proteins of CHEN-apo. We will describe the projection procedure.

Let H be a protein structure from CHEN-holo, $L_1, \cdots, L_k$ be ligands within H, and P be the structure of the corresponding protein in CHEN-apo. We positioned the ligands L_i onto P as follows.

- Find a sequence alignment of H and P, using the software MMseqs2 ([14]).
- Find a SE(3) transformation ϕ_i from the space of H onto the space of P that minimizes the mean of squared distances between the alpha carbons of the pairs of residues (R, R') such that
  - R is from H and R' is from P
  - R and R' are aligned
  - R or one of its (two) neighboring residues is within 4Å from L_i.

  (If there was no such pair, the ligand was ignored, and not projected to the apo structure)
- Consider $L'_i = \phi_i(L_i)$ as a ligand within the structure P

2.4. **Dataset profiles.** The procedures described in the previous two sections resulted in the following dataset profiles:

TABLE 1. Dataset profiles

dataset	number of proteins	number of binding sites	average number of binding sites	maximum number of binding sites
scPDB	16612	17594	1.06	4
COACH420	299	494	1.65	12
HOLO4K	3378	7141	2.11	24
CHEN-holo	104	244	2.35	11
CHEN-apo	104	243	2.34	11

3. Comparison of our evaluation metrics with those of baseline methods

As explained in 3.7 of the manuscript, we used three evaluation metrics for BSP — *success rate*, *IOU*, and *conditional IOU*. These metrics are inspired by those in previous works, but there are some differences.

3.1. **Success rate.** This metric is similar to those used in the baseline methods ([1], [15] and [10]). Compared to their definitions, our definition makes two specific choices. First, as in [15], our definition uses F1 score to compare predicted binding sites and actual ligands in each protein. This differs from the *precision* version used in [1] and [10]. These two may differ when more than one *ligand of interest* is bound to the protein. In that case, the *precision* version may give the *perfect* score when all predicted binding sites are nearby a single ligand. Secondly, in our definition, the definition of "detection" is based on DCA (used in [1] and [10]), instead of Distance from Center to Center (DCC, used in [1] and [15]). DCC, unlike DCA, computes the distance between the predicted binding site center and the center of the binding pocket associated to the ligand. Although this may be semantically more adequate than DCA, DCC has a critical limitation—it can be measured only when the binding pocket associated to the ligand is manually annotated or extracted successfully by a computational means. Indeed, in [15], this fact limited the evaluation on a non-annotated dataset to the ligands whose associated binding pockets could be successfully extracted by an external software.

3.2. **IOU.** This metric is analogous to a metric used in [15] to evaluate the *segmentation* ability of the model, but differs from it in two aspects. Firstly, we defined the "closest ligands" in terms of DCA, instead of DCC. Secondly, we compared the shape of the predicted binding site and the actual binding site in terms of the protein residues, instead of the segmented 3D volume as in [15]. The last choice reflects our emphasis on the task of BRI, which has its own applications that cannot be accomplished by volume segmentation, as explained in the introduction.

3.3. **Conditional IOU.** This metric is analogous to a metric used in [1]. However, their version computed the IOU out of predictions made from Fpocket-generated binding sites that are closest to the ligands, instead of binding sites that their BSD model predicted. Although this is a reasonable approach, other baseline methods that are not based on Fpocket cannot be assessed in this way.

Note that DeepSurf [10] also used a similar metric to evaluate models' binding site segmentation capability conditional on the successful location of the binding sites.

4. Effects of Mutation on Model Performance

Mutations can influence ligand binding in several ways. They can either maintain or disrupt the binding and can also introduce or eliminate a residue's involvement in the binding process ([12], [16], [9]). Understanding whether our BSD and BRI models can detect these changes is crucial for both a detailed evaluation of their performance and practical applications such as drug selectivity. In particular, our homology-based augmentation process may pose a question as to whether it leads to models' insensitivity to mutations.

We examined two types of proteins for our analysis: HSP90 ([9]) and HIV protease 1 ([16]). Both cases involved mutations that preserved the binding sites. In both cases, the reference protein was in our train set and only the mutants not in the train set were examined.

We evaluated the predictions made by our models (both the full models and the models trained without the homology augmentation) on the reference proteins and the mutants. In addition to measuring the DCA of the top-scored pocket center (result of BSD) and the IOU of the BRI predictions on the top-scored pocket, we tried to measure the BRI model's tendency to identify residues selectively for the references and the mutants. More specifically, we counted the residues in the following classes:

- **C1**: Predicted binding residues in the mutant that did not correspond to a predicted binding residue in the reference.
- **C2**: Predicted binding residues in the reference that did not correspond to a predicted binding residue in the mutant.
- **C3**: False positive residues in the mutant that correspond to a true binding residue in the reference.
- **C4**: False positive residues in the reference that correspond to a true binding residue in the mutant.

Table 2. Predictions made by our main models on proteins with conservative mutations

Protein	Type	PDB	Location	ligand	1st pocket DCA	Residue recall	FP	C1	C2	C3	C4
HSP90	ref	1YET	scPDB train	GDM	1.4Å	17/17	3	-	-	-	-
HSP90	mut	2YGE	not in scPDB	GDM	0.8Å	16/16	3	0	1	1	0
HIV-P	ref	1DMP	scPDB train	DMQ	0.7Å	27/27	3	-	-	-	-
HIV-P	mut	1A9M	scPDB test	U0E	1.2Å	21/21	9	0	0	8	2
HIV-P	mut	3A2O	scPDB test	KNJ	1.6Å	26/26	4	0	0	3	2

Table 3. Predictions made by our models trained without the homology augmentation on proteins with conservative mutations

Protein	Type	PDB	Location	ligand	1st pocket DCA	Residue recall	FP	C1	C2	C3	C4
HSP90	ref	1YET	scPDB train	GDM	1.4Å	**16**/17	3	-	-	-	-
HSP90	mut	2YGE	not in scPDB	GDM	0.8Å	16/16	**5**	**3**	1	**0**	0
HIV-P	ref	1DMP	scPDB train	DMQ	0.7Å	**26**/27	3	-	-	-	-
HIV-P	mut	1A9M	scPDB test	U0E	1.2Å	21/21	9	**1**	0	8	2
HIV-P	mut	3A2O	scPDB test	KNJ	1.6Å	26/26	4	**1**	0	3	2

The results are summarized in Table 2 and Table 3. In all cases, the top-scored pocket centers predicted by the BSD models were sufficiently close to the provided ligands.

While both BRI models correctly identified most of the true binding residues, there were a few false positives. Many of the false positives turned out to be true binding residues of the counterpart (the reference for the mutants and the mutant for the references), especially in the case of HIV protease 1, as seen from the columns **C3** and **C4**.

The homology augmentation reduced most discrepancies between the binding residue predictions on the references and those on the mutants, as seen from the columns **C1** and C2. This was helpful in the case of HSP90 mutant, as it eliminated three existing false positives while introducing only one new one.

In summary:

- Our BSD model maintained its performance for non-trained mutants, with or without homology augmentation.
- Our BRI model mostly maintained its performance for non-trained mutant, with or without homology augmentation.

- Our BRI model tends to make synchronized predictions between homologues, where corresponding residues tend to be simultaneously predicted as binding or non-binding residues.
- The homology augmentation may lead to tighter synchronization.
- The effect of synchronisation is ambiguous. While it may introduce false positives for non-trained proteins in some cases, further tightening it through the homology augmentation can be helpful in other cases.

5. Rationales behind numerical criteria

Throughout our method, several choices were made about the exact numerical criteria. In this section, we elucidate the rationales behind them.

5.1. **The input range.** Our BRI and BSD modules take residues with alpha carbons within 17Å from the candidate binding site as inputs. This can be explained in two ways—computational demand and comparison with Deeppocket. Firstly, it should be noted that an ideal input radius should encompass all ground truth binding residues in most cases. Note that the third quartile (among the positive pockets in scPDB train set) value of the maximum distance between the pocket center and the alpha carbon of a binding residue was 18.5Å. We took the largest value that was deemed feasible with our computational resources, which was 17Å. Another reason was that the value 17Å was comparable with the input range of Deeppocket's segmentation model. Deeppocket's segmentation model takes cubical grids of half side length 16Å as inputs, which implies that residues with alpha carbon barely outside this range can be potentially identified as binding residues, even with the worst rotation. Therefore, our BRI model's input range can be seen as just as large as Deeppocket's input range.

5.2. **The lower threshold for the distance between proxy centers and pocket centers.** Our homology-based augmentation labels a candidate binding site found by Fpocket as "positive" if it is within 7.5Å from a "proxy center" (the center of mass of alpha carbons) of a sequence fragment homologous to a known binding site. Roughly speaking, 7.5Å was set such that most known binding sites would have been determined as "positive" if the binding residues had been perfectly aligned (by MSA) with another known binding site (say itself). More specifically, we took all pairs of pocket centers and associated ligands (DCA ¡ 4Å) from the scPCB train set, computed the distances from the pocket centers to the "proxy centers" of the ligand-bound residues, and computed the value at the top-5% quantile to get 7.5Å.

5.3. **The upper threshold for the distance between proxy centers and pocket centers.** Our homology-based augmentation labels a candidate binding site found by Fpocket as "negative" if it is outside 30Å from all proxy centers of sequence fragments homologous to known binding sites. In deciding this upper threshold, we took an approach that is similar to the lower threshold but rather conservative. Namely, we set the value such that the presence of a binding-site-homologous residue outside this range is extremely unlikely to be evidence that the potential binding site is an actual binding site (hence the condition that all proxy centers are beyond 30Å implies that there is no evidence by homology at all). More specifically, we took all pairs of pocket centers and associated ligands (DCA ¡ 4Å) from the scPCB train set, computed the maximum distances from the pocket centers to the alpha carbons of the ligand-binding residues, and computed the value at the top-0.2% quantile to get 30Å(The maximum was 37.6Å).

References

[1] R. Aggarwal, A. Gupta, V. Chelur, C. Jawahar, and U. D. Priyakumar. Deeppocket: ligand binding site detection and segmentation using 3d convolutional neural networks. *Journal of Chemical Information and Modeling*, 2021.

[2] A. Ahmed, R. D. Smith, J. J. Clark, J. B. Dunbar Jr, and H. A. Carlson. Recent improvements to binding moad: a resource for protein–ligand binding affinities and structures. *Nucleic acids research*, 43(D1):D465–D469, 2015.

[3] K. Chen, M. J. Mizianty, J. Gao, and L. Kurgan. A critical comparative assessment of predictions of protein-binding sites for biologically relevant organic compounds. *Structure*, 19(5):613–621, 2011.

[4] J. Desaphy, G. Bret, D. Rognan, and E. Kellenberger. sc-pdb: a 3d-database of ligandable binding sites—10 years on. *Nucleic acids research*, 43(D1):D399–D404, 2015.

[5] F. Fuchs, D. Worrall, V. Fischer, and M. Welling. Se (3)-transformers: 3d roto-translation equivariant attention networks. *Advances in Neural Information Processing Systems*, 33:1970–1981, 2020.

[6] J. Jumper, R. Evans, A. Pritzel, T. Green, M. Figurnov, O. Ronneberger, K. Tunyasuvunakool, R. Bates, A. Žídek, A. Potapenko, et al. Highly accurate protein structure prediction with alphafold. *Nature*, 596(7873):583–589, 2021.

[7] R. Krivák and D. Hoksza. P2rank: machine learning based tool for rapid and accurate prediction of ligand binding sites from protein structure. *Journal of cheminformatics*, 10(1):1–12, 2018.

[8] I. Loshchilov and F. Hutter. Sgdr: Stochastic gradient descent with warm restarts. *arXiv preprint arXiv:1608.03983*, 2016.

[9] S. H. Millson, C.-S. Chua, S. M. Roe, S. Polier, S. Solovieva, L. H. Pearl, T.-S. Sim, C. Prodromou, and P. W. Piper. Features of the streptomyces hygroscopicus htpg reveal how partial geldanamycin resistance can arise with mutation to the atp binding pocket of a eukaryotic hsp90. *The FASEB Journal*, 25(11):3828–3837, 2011.

[10] S. K. Mylonas, A. Axenopoulos, and P. Daras. Deepsurf: a surface-based deep learning approach for the prediction of ligand binding sites on proteins. *Bioinformatics*, 37(12):1681–1690, 2021.

[11] RDK. p2rank-dataset. `https://github.com/rdk/p2rank-datasets`, 2017.

[12] T. Ridky and J. Leis. Development of drug resistance to hiv-1 protease inhibitors. *Journal of Biological Chemistry*, 270(50):29621–29623, 1995.

[13] P. Schmidtke, C. Souaille, F. Estienne, N. Baurin, and R. T. Kroemer. Large-scale comparison of four binding site detection algorithms. *Journal of chemical information and modeling*, 50(12):2191–2200, 2010.

[14] M. Steinegger and J. Söding. Mmseqs2 enables sensitive protein sequence searching for the analysis of massive data sets. *Nature biotechnology*, 35(11):1026–1028, 2017.

[15] M. M. Stepniewska-Dziubinska, P. Zielenkiewicz, and P. Siedlecki. Improving detection of protein-ligand binding sites with 3d segmentation. *Scientific reports*, 10(1):1–9, 2020.

[16] I. T. Weber and J. Agniswamy. Hiv-1 protease: structural perspectives on drug resistance. *Viruses*, 1(3):1110, 2009.

[17] J. Yang, A. Roy, and Y. Zhang. Protein–ligand binding site recognition using complementary binding-specific substructure comparison and sequence profile alignment. *Bioinformatics*, 29(20):2588–2595, 2013.
